# The Dual Role of Kinin/Kinin Receptors System in Alzheimer’s Disease

**DOI:** 10.3389/fnmol.2019.00234

**Published:** 2019-10-01

**Authors:** Bingyuan Ji, Qinqin Wang, Qingjie Xue, Wenfu Li, Xuezhi Li, Yili Wu

**Affiliations:** ^1^Neurobiology Institute, School of Mental Health, Jining Medical University, Jining, China; ^2^Department of Pathogenic Biology, Jining Medical University, Jining, China; ^3^Shandong Collaborative Innovation Center for Diagnosis, Treatment and Behavioral Interventions of Mental Disorders, Institute of Mental Health, Jining Medical University, Jining, China; ^4^Shandong Key Laboratory of Behavioral Medicine, School of Mental Health, Jining Medical University, Jining, China

**Keywords:** Alzheimer’s disease, kinins receptors, amyloid beta, neuroinflammation, neuroprotection

## Abstract

Alzheimer’s disease (AD) is the most common neurodegenerative disease characterized by progressive spatial disorientation, learning and memory deficits, responsible for 60%–80% of all dementias. However, the pathological mechanism of AD remains unknown. Numerous studies revealed that kinin/kinin receptors system (KKS) may be involved in the pathophysiology of AD. In this review article, we summarized the roles of KKS in neuroinflammation, cerebrovascular impairment, tau phosphorylation, and amyloid β (Aβ) generation in AD. Moreover, we provide new insights into the mechanistic link between KKS and AD, and highlight the KKS as a potential therapeutic target for AD treatment.

## Introduction

Kinins are a group of peptides derived from kininogens, affecting the control of blood pressure, vascular permeability and pain transmission through activating two G protein-coupled receptors (GPCRs), namely bradykinin B1 receptor (B1R) and bradykinin B2 receptor (B2R). B2R is constitutively expressed in many tissues under normal conditions and displays higher affinity for bradykinin (BK) and kallidin peptides, while B1R is expressed during pathological states and binds Des-Arg^9^-BK and Leu^8^-des-Arg^9^-BK. In the central nervous system (CNS) of mammals, B2R is expressed in the cerebral cortex, ependyma, thalamus, basal nuclei and hypothalamus, whereas B1R is present in the entorhinal cortex, spinal cord and dentate gyrus. In addition to neurons and astrocytes, microglia also express bradykinin receptor (BR; Noda et al., 2004).

**Figure 1:**
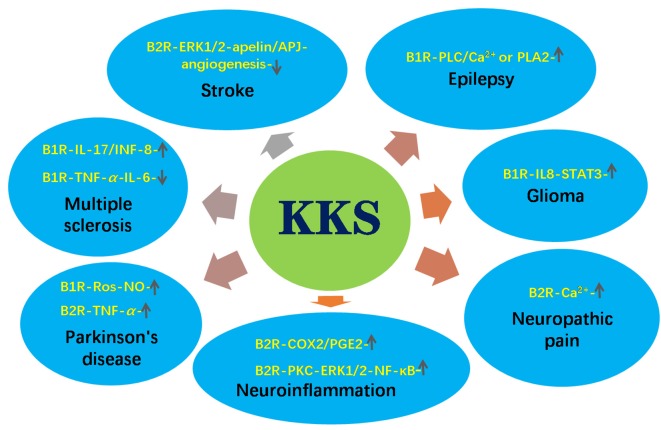
The roles of kinin/kinin receptors system (KKS) in neurological diseases. The upward arrow indicates the pathways promoting the pathogenesis of the diseases. The downward arrow indicates the pathways inhibiting the pathogenesis of the diseases.

As a neuropeptide, BK and its receptors play an important role in many neurological diseases. As reported in our previous review (Ji et al., 2017), kinin/kinin receptors system (KKS) may play a neuroprotective role in cerebral ischemia and stroke through triggering extracellular regulated protein kinases1/2 (ERK1/2)-apelin/APJ signaling pathways, which plays a key role in ischemia-reperfusion injury (Qiu et al., 2017; Wu et al., 2017, 2018). Xia et al. (2006) found that the infarction area in B2R knockout mice was larger than that in wild-type mice, suggesting that B2R protects against ischemia-reperfusion injury in brains. However, KKS can also aggravate ischemia-reperfusion injury. Growing evidence shows that KKS regulates neurogenesis, cerebrovasculogenesis (Alexander-Curtis et al., 2019) and other neurophysiological processes and is involved in neurological disorders (Figure 1). For example, B2R may promote epileptogenesis through increasing hippocampal excitability in mice (Rodi et al., 2013). It has been demonstrated that B1R can activate myosin phosphorylation and phospholipase C, which leads to elevated intracellular Ca^2+^ level and induces seizures. In addition, B1R and B2R activate phospholipase A2 to produce prostaglandins, which leads to epilepsy and brain edema after seizures. In human glioblastoma cell lines, BK increases the expression levels of STAT3 and SP-1 *via* B1R, causing interleukin-8 (IL-8) expression and cell migration (Liu et al., 2019). In brain astrocytes, BK activated protein kinase C (PKC)/ERK1/2/nuclear factor kappa B (NF-κB) signaling pathway, which up-regulated inducible nitric oxide synthase (iNOS) expression, resulting in neuroinflammation (Hsieh et al., 2006). B2R can be activated by dynorphin to maintain neuropathic pain (Lai et al., 2006).

Kinin peptides were also involved in neurodegenerative diseases such as Parkinson’s disease (PD), multiple sclerosis (MS) and Alzheimer’s disease (AD). Niewiarowska-Sendo et al. (2016) found that the B1R agonist des-Arg^10^-kallidin enhanced reactive oxygen species (ROS) and NO levels and accelerated PD process (Niewiarowska-Sendo et al., 2016), while the B2R agonist BK induced the production of tumor necrosis factor-α (TNF-α), leading to cell death in PD (Niewiarowska-Sendo et al., 2016). MS is also tightly related to KKS. B1R agonist R38 inhibited T lymphocyte into the CNS in MS mice (Schulze-Topphoff et al., 2009). However, in another study, the activation of B1R promoted Th17 lymphocytes across the blood brain barrier (BBB; Göbel et al., 2011), indicating its double role in the development of MS. Recent studies indicated that KKS is implicated in the pathogenesis of AD through various mechanisms and might be a potential target for AD treatment. Therefore, we aim to summarized the roles of KKS in the pathogenesis of AD and provide novel insights into the therapeutic potential for AD treatment by targeting KKS.

## Alzheimer’s Disease

AD is a neurodegenerative disorder characterized by progressive memory deficits, weakness of intellectual capacity and behavioral abnormalities. Senile plaques, loss of synapses and neurofibrillary tangles are the pathological features of AD (Wu et al., 2014a,b; Zhang S. et al., 2018; Qiu et al., 2019). The major component of neuritic plaques is the amyloid β (Aβ) protein, a self-aggregating peptide of 36–43 amino acids. As a key player in the cognitive impairment of AD (LaFerla et al., 2007), Aβ is produced by sequential enzymatic cleavage of amyloid precursor protein (APP) by β- and γ-secretases (Selkoe, 2001; Ly et al., 2013; Wang et al., 2017, 2019; Zhang S. et al., 2018). Pathological mutations in *APP* gene, *PSEN1* gene or *PSEN2* gene, *APP* duplication and trisomy of chromosome 21 promote Aβ production and neuronal apoptosis contributing to the pathogenesis of early-onset AD (Steiner et al., 1998; Wu and Song, 2013; Sun et al., 2014; Wu et al., 2015; Zhang et al., 2017; Zhang S. et al., 2018). Moreover, the dysregulation of TMP21, a γ-secretase modulator, also contributes to increased Aβ generation in AD (Chen et al., 2006; Zhang X. et al., 2018; Zhang et al., 2019).

Growing evidence indicates that neuropeptides and their receptors are implicated in the pathogenesis of AD (Qiu et al., 2017; Wu et al., 2017, 2018; Aminyavari et al., 2019). Strikingly, brain regions (e.g., hippocampus, cerebral cortex) that are affected early during AD are also the regions where bradykinin and their receptors are prominently expressed. Several pieces of evidence suggests that KKS might play an important role in the pathogenesis of AD (Viel and Buck, 2011), for example, the cleavage of high molecular weight kininogens was observed in the cerebrospinal fluid (CSF) of AD patients (Kasuya et al., 1988). After the rats were treated with chronic cerebroventricular infusion of Aβ, BK levels were increased in the brain and CSF, as well as increased densities of B1R and B2R in different brain areas such as the prefrontal cortex and hippocampus (Iores-Marçal et al., 2006; Prediger et al., 2008; Viel et al., 2008). Moreover, the treatment of human skin fibroblasts with BK increased the release of secreted APP β (sAPPβ), which is blocked by the selective B2R antagonist HOE140. Thus, a “vicious circle” was formed, and it may be responsible for the later cognitive deficits. Indeed, Prediger et al. (2007) found that low dose of BK injection into rat hippocampus resulted in learning and memory decline (Wang and Wang, 2002), possibly due to the reduction in synaptic density (Medeiros et al., 2007). However, pharmacological blockade or genetic deletion of the B1R and B2R significantly reverses the cognitive impairments caused by a single intracerebroventricular (i.c.v) injection of Aβ1–40 in rodents, suggesting that B1R and B2R were potential drug targets for the treatment of AD (Prediger et al., 2008). On the other hand, B1R blockade protects the brain from damage in mice by controlling BBB leakage (Raslan et al., 2010), which is associated with AD (Zipser et al., 2007).

However, various studies have shown that kinin receptors also play neuroprotective roles in neurodegenerative diseases. For example, the absence of B2R decreased aversive-related memory and neuronal density, suggesting that the lack of B2R could contribute to loss of memory and neurodegeneration (Caetano et al., 2015).

## Mechanisms of Kinin/Kinin Receptors System in Alzheimer’s Disease

### Kinin/Kinin Receptors System Mediates Neuroinflammation in Alzheimer’s Disease

Mitogen-activated protein kinases (MAPKs) and their downstream transcription factors such as NF-κB contributes to the maintenance of neuroinflammation (Medeiros et al., 2010). Activation of this signaling pathway leads to the release of cyclooxygenase-2 (COX-2), TNF-α, IL1-β, inducible and neuronal nitric oxide synthase (iNOS and nNOS) isoforms and BK (Passos et al., 2010). Increasing evidence demonstrates the mediatory role of kinin-kallikrein system in the brain edema, ischemia and cytokine release events that promote inflammation (Su et al., 2009). BK and des-Arg^9^-BK are often regarded as inflammatory mediators in the CNS (Viel and Buck, 2011). Once stimulated, B1Rs and B2Rs lead to the elevation of intracellular Ca^2+^, arachidonic acid release, and the production of some inflammatory mediators, such as prostanoids, tachykinins, cytokines and NO. Of interest, B2R mainly induced the acute inflammatory process, while chronic inflammation is mainly attributed to B1R. After agonist binding, B1R is desensitized partially, whereas B2R is quickly desensitized. Moreover, kinins-mediated inflammatory response is also closely related to microbial infection (Karkowska-Kuleta et al., 2010; Rapala-Kozik et al., 2010).

It is well known that Aβ triggers a strong neuroinflammation process for the accumulation of activated astrocytes and microglia surrounding senile plaques (Salminen et al., 2009; Alam et al., 2016). The activation of astrocytes and microglia produces abundant inflammatory mediators. Aβ increases the secretion of iNOS (Diaz et al., 2011; Ferretti et al., 2012) and some pro-inflammatory cytokines such as NO and TNFα (Tai et al., 2015). Furthermore, treatment of aggregated Aβ1–40 in mice may result in oxidative stress and memory deficits (Prediger et al., 2007). Meanwhile, these inflammatory proteins upregulate the expression of APP, resulting in increased Aβ production, and finally aggravate the learning and memory deficits(Yirmiya and Goshen, 2011; Barrientos et al., 2015). Interestingly, Bicca et al. (2015) found that pretreatment with B2R antagonist HOE140 significantly attenuated neuroinflammation induced by Aβ in mice through reducing the activation of microglial and the expression of inflammatory factors. Moreover, B2R antagonist also inhibits the activation of NF-κB and MAPKs, confirming that the pathways depended on the activation of B2R. B2R antagonist HOE140 significantly decreases synaptophysin and PSD-95 in the hippocampus of mice treated with Aβ1–40. Thus, the anti-inflammatory effect of B2R antagonist prevents synaptic loss and cognitive deficits induced by Aβ1–40 in mice. In addition, B1R also exerts an important role in inflammation-related conditions, such as AD. Blockade of B1R significantly improves cognitive function in AD mice (Amaral et al., 2010; Passos et al., 2013). Taken together, Aβ peptide can induce variable inflammatory mechanisms which can be enhanced by kinins and their receptors, suggesting that KKS plays a key role in inflammation in the central and in the periphery nervous system.

### Kinin/Kinin Receptors System Regulates Glial Cells Activation in Alzheimer’s Disease

Microglia, the resident macrophages of the CNS, is involved in neuronal cell defense from extremely harmful stimuli and capable of protecting cells from injury or death (Fu et al., 2014). Microglial cells might contribute to the removing of Aβ deposits in AD mouse brain (Van Nostrand et al., 2012). The inactivation of microglial increased Aβ deposition in the hippocampus. B1R protein levels were mainly upregulated in astrocytes around Aβ plaques in the brain. B1R up-regulation induces the accumulation of activated glial cells, which can reduce Aβ deposition through phagocytic-dependent clearance mechanism.

However, microglia can change its activation to neurotoxic phenotype, and these changes were also reported in AD mice (McGeer et al., 1988; Akiyama et al., 2000; Perez-Polo et al., 2015). Once activated, microglia can release various chemokines, complement proteins, free radicals, and proinflammatory cytokines, which affect the removing of Aβ during the development of AD (Wang et al., 2015; Zamberletti et al., 2015). Accumulation of Aβ induces neuroinflammation through binding with microglial innate GPCRs, which is postulated to induce an amyloid cascade-inflammatory hypothesis of AD. In AD model mice, B1R antagonist SSR240612 not only reduced microglial activation, diffuse and dense-core Aβ plaques, brain levels of soluble Aβ1–42, but also remarkably improved spatial learning and memory. Moreover, continuous treatment with SSR240612 normalized the levels of memory-related protein Egr-1 in the dentate gyrus (Lacoste et al., 2013). Cerebral blood flow (CBF) responses and cerebrovascular dilations were balanced with prolonged B1R antagonist treatment, and the levels of endothelial nitric oxide synthase (eNOS) and B2R protein was also normalized. In addition, activated microglia and astrocytes express inflammatory molecules in Tg-SwDI mice brains, closely related to cerebral microvascular amyloid (Fan et al., 2007). However, B1R antagonism R715 treatment reduces the activation of the proinflammatory factor NF-κB through reducing glial cell activation (Passos et al., 2013). Taken together, these findings demonstrate that B1R plays a vital role in AD pathogenesis, possibly through the modulation of activated glial cell accumulation. Therefore, the regulation of B1R activation may represent a new therapeutic target for AD.

### Kinin/Kinin Receptors System Promotes Tau Protein Phosphorylation in Alzheimer’s Disease

Another key protein related to AD is the microtubule associated protein tau (MAPT), which can be phosphorylated at Ser/Thr. Hyperphosphorylated MAPT causes microtubule destabilization and neurofibrillary tangles, finally resulting in impaired protein transport (Fleisher-Berkovich et al., 2010). Interestingly, tau protein phosphorylation and abnormal behaviors were observed after BK injection into the rat hippocampus (Wang and Wang, 2002). Similar results have been observed in skin fibroblasts of patients with Down’s syndrome or pathogenic PSEN1 mutations, in which treatment with BK induced the selective phosphorylation of tau protein on Ser residues (Jong et al., 2003). However, this phosphorylation was not found in unaffected age-matched control human skin fibroblasts. Further studies demonstrated that BK-induced phosphorylation of tau protein is blocked by the PKC inhibitor GF109203X in Trisomy 21 fibroblasts and in PS-1 mutant fibroblasts, indicating that the BK-induced tau phosphorylation is mediated through activation of PKC. In addition, the growth regulator cyclin-dependent kinases cdk2 and cdk5 and MAPK family members are also candidate kinases that can phosphorylate tau protein at particular Ser residues, promoting tangle formation. Finally, abnormally enhanced and sustained ERK1/2 activation in response to BK stimulation has been reported in familial and sporadic AD skin fibroblast lines, causing tau protein phosphorylation (Zhao et al., 2002). ERK1/2 phosphorylates tau at Ser-262 and Ser-356, which are in the microtubule-binding regions of tau protein. Phosphorylation of tau protein at Ser-262 helps to assemble and stabilize microtubules. Thus, genetic deletion or the pharmacological inhibition of B2R may rescue the cognitive deficits in Aβ1–40-treated mice through blocking or reducing tau hyperphosphorylation.

### Cerebrovascular Dysfunction Mediated by Kinin/Kinin Receptors System

A growing number of studies have shown that cerebralvascular injury occurs in the early stage of AD (Nicolakakis and Hamel, 2011), and Aβ not only alters neuronal function but promotes cerebrovascular dysfunction (Nelson et al., 2016). Faulty Aβ clearance is thought to induce elevated brain Aβ levels, rather than increased Aβ production. The predominant receptor mediating clearance of Aβ is the lipoprotein receptor-related protein-1 (LRP-1), which is expressed mainly at the abluminal side of the BBB. In normal aging and AD patients, the expression levels of LPR-1 decreased significantly in brain endothelial cells (Donahue et al., 2006), whereas B1R antagonist SSR240612 increased LRP-1 protein levels in cerebral microvessels (Lacoste et al., 2013).

Moreover, elevations of brain Aβ levels not only impaired selected vasodilatory responses of the cerebral circulation but also reduced resting CBF in APP mice and in AD patients (Roher et al., 2012; Iadecola, 2013). However, SSR240612 treatment significantly improved these hemodynamic responses (Lacoste et al., 2013). In AD model mice, vasoconstrictions were unaltered up to 21 months of age, whereas the dilatations to ACh and calcitonin gene-related peptide (CGRP) was impaired early (Niwa et al., 2002; Tong et al., 2005). Surprisingly, impaired cerebrovascular dilation mediated by ACh was rescued after SSR240612 treatment for 5 or 10 weeks as well as dose-dependent and maximal responses. Consistently, SSR240612 administration reduced the levels of B2R and eNOS proteins in cerebral arteries in APP mice, improving learning and memory abilities (Lacoste et al., 2013). Thus, SSR240612 might have a potential neuroprotective effect for AD.

### Kinin/Kinin Receptors System in Metabolic Abnormalities in Alzheimer’s Disease

Similar to AD, diabetes mellitus is a prominent chronic health condition in older persons. Increasing evidences showed diabetes shared some common pathogenic mechanisms with AD such as metabolic abnormalities, endoplasmic reticulum (ER) stress, and impaired insulin signaling [e.g., phosphatidylinositol 3 kinase (PI3K)-glycogen synthase kinase 3β (GSK3β) signaling; Kuljiš and Salković-Petrišić, 2011; Blázquez et al., 2014; Fernando et al., 2017; Vieira et al., 2018]. Moreover, as pathological hallmarks of AD, Aβ polypeptide deposition exists not only in the brain of AD patients, but also in the pancreatic islets of AD mice and diabetes patients (de Nazareth, 2017; Lu et al., 2018), leading to deficits in glucose utilization. Indeed, type 2 diabetes is strongly associated with cognitive dysfunction and significantly increases the risk of dementia (Cha et al., 2014). Recently, AD is regarded as “Type-3-Diabetes” (Ahmed et al., 2015; Kandimalla et al., 2017) for AD possess both insulin deficiency and insulin resistance, features of type 1 diabetes and type 2 diabetes respectively. These characteristics cause impaired glucose metabolism, which in turn lead to pro-apoptosis and APP-Aβ cascades in AD (Dhamoon et al., 2009).

Growing evidence showed that kinins and their receptors were involved in the glucose homeostasis and abnormal metabolism, which contributes to metabolic syndrome including obesity, diabetes mellitus, and hypertension (Abe et al., 2007; Morais et al., 2015; Talbot et al., 2016; Sales et al., 2019). Isami et al. (1996) found that bradykinin enhanced insulin-mediated glucose uptake through acting the B2R. Similarly, the B1R agonist des-Arg^9^-bradykinin can induce the release of insulin and increase pancreatic vascular permeability, suggesting an important role for B1R in the pathophysiology of diabetes and related diseases such as AD (Araujo et al., 2006). On the contrary, several studies have demonstrated that the blockade of B1R and B2R with selective antagonists could prevent hyperglycemia and insulitis through regulating glucose uptake (Zuccollo et al., 1999; Beard et al., 2006; Talbot et al., 2016). Taken together, the role of the KKS in glucose uptake and related diseases remains controversial.

### Neuroprotective Role of Kinin Receptors in Alzheimer’s Disease

Although BK is regarded as a key proinflammatory factor, it has been shown that BK has anti-inflammatory effects in brain (Viel and Buck, 2011; Ji et al., 2017). For example, Noda et al. (2007) found that the B1R agonist inhibited LPS-induced cytokines release from microglia but was substantially canceled by B1R antagonist. Other groups showed B1R agonist reduced prostaglandin E2 (PGE2) synthesis in glial cells under both LPS-induced and non-stimulated conditions (Levant et al., 2006), leading to anti-inflammatory effect. Administration of B1R antagonist R-715 significantly increased TNF-α and NO release from BV2 cells, and also increased iNOS expression levels, suggesting an anti-inflammatory role for B1R (Asraf et al., 2016, 2017). Moreover, inhibiting B1R with antagonists to 8-months old triple mutant APP mice increased fibrillar Aβ deposition, particularly in the dentate gyrus, subiculum, cortex and thalamus, although Aβ production and cognitive function were not changed (Passos et al., 2013).

Likewise, B2R upregulated mRNA of nerve growth factor in astrocytes (Noda et al., 2007), establishing a neuroprotective condition. Caetano et al. found there were more amyloid plaques in Aβ-injected B2RKO mice than that in wild-type mice, suggesting a neuroprotective role for B2R (Caetano et al., 2015). Subsequently, the same group demonstrated that B2R binding is increased in B1R knockout (B1RKO) Aβ mice (Caetano et al., 2015), and elevated B2R density contributes to memory preservation in Aβ-injected mice lacking B1R (Lacoste et al., 2013). In support of these data, B2RKO mice have been reported to exhibit memory retention deficits compared with wild-type mice (Lemos et al., 2010). Similar results were observed in B2RKO C57Bl6 mice, where mice treated with Aβ showed a significant reduction in memory consolidation (Viel and Buck, 2011). Further studies show that the dose-dependent stimulation of N9 and BV2 cell lines or primary microglial cells with BK enhanced the removal of Aβ deposit (Fleisher-Berkovich et al., 2010). Moreover, AD patients have altered copper levels, and copper binding can hinder the oligomerization of BK, which in turn reduces IL-1β expression and ERK1/2 phosphorylation (Naletova et al., 2016).

## Kinin Receptors-Induced Signaling Cascades in Alzheimer’s Disease

### iNOS/NO Signaling Pathway

The main characteristic of neuroinflammation is the production of excessive proinflammatory factors, such as ROS and NO (Block and Hong, 2005). Specifically, NO is usually regarded as a highly toxic molecule. As mentioned above, high concentration of NO cause neuron death and CNS tissue injury and it can accelerate the progression of AD (Nakamura and Lipton, 2011). In contrast, Sarit et al. (2012) found that BK decreased LPS-induced NO and TNF-α synthesis in activated microglial cells, due to the inhibition of NF-κB activity by BK. Also, BK reduced the expression of iNOS in BV2 and N9 microglial cells (Ben-Shmuel et al., 2013), which increased the phagocytosis of aggregated Aβ, suggesting that BK plays a role in Aβ clearance from brain tissue and regulates brain response to neuroinflammation (Fleisher-Berkovich et al., 2010).

In the brain, intracellular cAMP can induce microglia activation, significantly increasing Aβ-induced NO release *via* induction of iNOS (LaDu et al., 2001; Pannu and Singh, 2006). Interestingly, BK can inhibit PKA activity to reduce NO production in LPS-stimulated microglia cells. In addition, BK increases the Gαi protein in the plasma membrane followed by decreased levels of phosphorylated CREB. Thus, BK induced NO reduction in microglial involves inhibition of Gαs/cAMP/PKA/CREB signaling pathway.

### MEK/ERK1/2 Signaling Pathway

Several studies have shown that ERK1/2 signaling pathway is involved in the pathological process of AD. MEK/ERK1/2 has a high sensitivity and specificity in skin fibroblasts in the early stages of AD (Khan and Alkon, 2010). Thus, differential phosphorylation status of ERK1/2 may be a clinically powerful tool for early monitoring and diagnosis of AD. Moreover, this biomarker has a significant pathophysiological correlation with tau protein (Nokkari et al., 2018). The phosphorylation of tau protein may also be catalyzed by GSK3 and CaM Kinase II after treatment with BK in rat brain (Sengupta et al., 2006). In addition, BK enhances the expression of B2R and promotes the generation of inositol 1,4,5-trisphosphate (IP3). The elevation of ERK1/2 phosphorylation required activation of PKC as well as IP3-sensitive Ca^2+^ release and the non-receptor protein tyrosine kinase c-src after BK stimulation. Interestingly, the BK-induced ERK1/2 phosphorylation was not affected in the AD cells under PI3K blocker LY924002 treatment, suggesting that the ERK1/2 phosphorylation stimulated by BK appears to be independent of PI3K (Zhao et al., 2002).

However, ERK1/2 was also reported to be an endogenous negative regulator of AD, because it down-regulates the activity of γ- and β-secretase (Kim et al., 2006; Tamagno et al., 2009). In addition, the PKC has been implicated in increasing the sAPPα, and reducing amyloid plaque pathology in transgenic mice (Choi et al., 2006). The BK-induced increase of sAPPα release in PC-12 cells was mediated by PKC (Nitsch et al., 1998).

## Pharmacological Potential of KKS as a Therapeutic Agent for AD

Many GPCR agonists or antagonists have been reported as candidate drugs for the treatment of AD. For example, a serotonin type 6 receptor (5-HT6R) antagonist SB-742457 has been successfully used in phase I and phase II clinical trials and significantly improved the cognitive abilities of AD patients (Maher-Edwards et al., 2010, 2015; Callaghan et al., 2012). The M1 muscarinic receptor positive allosteric regulators MK-7622 can also effectively alleviate AD symptoms and have entered phase II clinical trials (Uslaner et al., 2018; Voss et al., 2018). Likewise, numerous studies showed that the KKS has huge implications in the treatment of neurological diseases (Scicli et al., 1984; Han et al., 2015; Niewiarowska-Sendo et al., 2016; González-Miguel et al., 2019). It has been demonstrated that B2R and B1R antagonists LF16-0687 and LF22-0542 could alleviate thermal hyperalgesia, promising their therapeutic potential for the treatment of neuropathic pain (Petcu et al., 2008). BRs blocker noscapine has been used as an antitussive drug due to its oral and low toxicity (Landen et al., 2004). Ni et al. (2019) further found that noscapine ameliorated cerebral vascular dysfunction in AD mouse using functional magnetic resonance imaging techniques (Ni et al., 2019). A variety of modified BR antagonists have shown potential in the treatment of AD. For example, selective BR antagonist DALBK reversed the spatial learning and memory deficits on 12-month-old rats (Bitencourt et al., 2017).

## Conclusions and Future Direction

**Figure 2:**
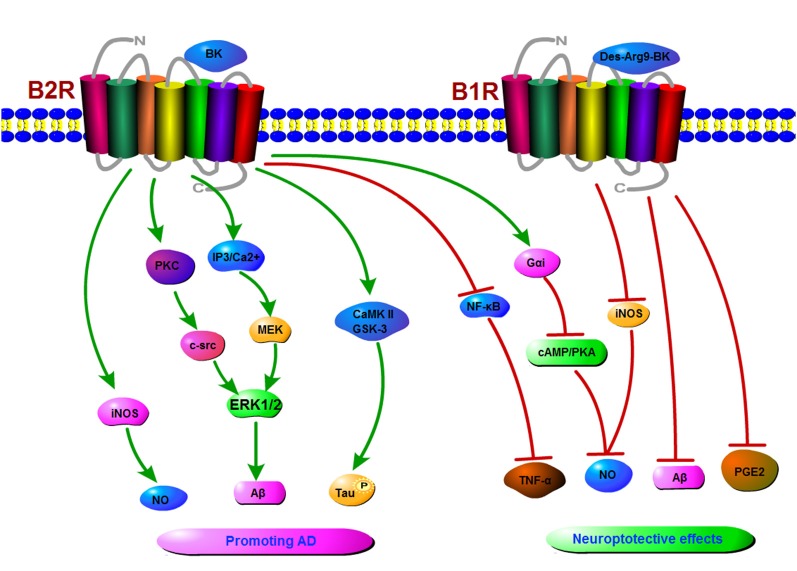
A schematic diagram of signaling pathways mediated by the KKS in Alzheimer’s disease (AD). The binding of kinins to B1R or B2R induces the activation of protein kinase C (PKC), inducible nitric oxide synthase (iNOS), extracellular regulated protein kinases1/2 (ERK1/2), CaMKII, ultimately resulting in an increase in NO, tau phosphorylation, and amyloid β (Aβ) production in AD. On the other hand, kinins receptors might play a neuroprotective role in AD by inhibiting the production of NO, Aβ, prostaglandin E2 (PGE2) etc.

KKS plays an important role in neurodegenerative processes like AD. Indeed, kinin and their receptors promote the development of AD through various molecular mechanisms including microglia activation, proinflammatory response, phosphorylation of tau protein and cerebrovascular impairment. However, BK is a double-edged sword which can play a neuroprotective role in AD through triggering multiple signaling pathways (Figure 2). Thus, kinin receptors could be a new target for treating AD. However, up to now, there is no definitive treatment option for AD, mainly because the pathogenesis of AD is not clear (Godyń et al., 2016; Hung and Fu, 2017).

## Author Contributions

BJ and QW performed the review and drafted the first version of the manuscript. QX, WL, XL and YW critically reviewed the manuscript for intellectual content. BJ, XL and YW revised the manuscript. All authors read and approved the final manuscript.

## Conflict of Interest

The authors declare that the research was conducted in the absence of any commercial or financial relationships that could be construed as a potential conflict of interest.

## Abbreviations

GPCR: G-protein-coupled receptor
AD: Alzheimer’s disease
B2R: bradykinin B2 receptor
B1R: bradykinin B1 receptor
BK: bradykinin
Aβ: amyloid β
APP: amyloid precursor protein
cAMP: cyclic adenosine monophosphate
CREB: cAMP-response element binding protein
KKS: kinin/kinin receptors system
eNOS: endothelial nitric oxide synthase
nNOS: neuronal nitric oxide synthase
iNOS: inducible nitric oxide synthase
COX-2: cyclooxygenase-2
TNF-α: tumor necrosis factor-α
ROS: reactive oxygen species
CNS: central nervous system
ERK1/2: extracellular regulated protein kinases1/2
GSK3: glycogen synthase kinase3
PI3K: phosphatidylinositol 3 kinase
IP3: inositol 1,4,5-trisphosphate
MAPK: mitogen-activated protein kinase
JNK: c-Jun N-terminal Kinase
p38: p38 MAPK
NF-κB: nuclear factor κB
PGE2: prostaglandin E2
BBB: blood brain barrier
PKC: protein kinase C
Src: short for sarcoma, member of the src family tyrosine kinases
HOE140: D-Arg-L-Arg-L-Pro-L-Hyp-Gly-L-(2-thienyl)Ala-L-Ser-D-1,2,3,4-tetrahydro-3-isoquinolinecarbonyl-L-(2α, 3β, 7aβ)-octahydro-1H-indole-2-carbonyl-L-Arg Icatibant acetate.
